# Trends in sex differences in the receipt of quality of care indicators among adults with diabetes: United States 2002-2011

**DOI:** 10.1186/s12902-017-0183-5

**Published:** 2017-06-06

**Authors:** Joni S. Williams, Kinfe G. Bishu, Alessandra St. Germain, Leonard E. Egede

**Affiliations:** 1 Department of Medicine, Medical College of Wisconsin, 9200 W. Wisconsin Avenue, Clinical Cancer Center Building Suite C5400, Milwaukee, WI 53226 USA; 2 Center for Patient Care and Outcomes Research (PCOR), Medical College of Wisconsin, Milwaukee, WI 53226 USA; 3 Division of General Internal Medicine, Center for Patient Care and Outcomes Research, Medical College ofWisconsin, 9200 W. Wisconsin Ave, Milwaukee, WI 53226 USA; 4 Center for Health Disparities Research, Department of Medicine, Medical University of South Carolina, 135Rutledge Avenue, Room 280, MSC 250593, Charleston, SC 29425 USA; 5 Department of Medicine, Division of General Internal Medicine and Geriatrics, Medical University of South Carolina, 171 Ashley Avenue, Charleston, SC 29425 USA

**Keywords:** Sex differences, Diabetes, Quality of care, Adults, Disparity

## Abstract

**Background:**

Evidence suggests disparities in quality of care (QoC) indicators based on sex exist in adults diagnosed with diabetes; however, this research is limited. Therefore, the objective of this research study was to assess differences in QOC indicators in a nationally representative sample of men and women with diabetes.

**Methods:**

Cross-sectional study of 17,702 men and women (≥18 years of age) with diabetes from the 2002–2011 Medical Expenditure Panel Survey Household Component. Sex was the main predictor variable, and the dependent variables were five binary indicators to measure QOC, which included testing of hemoglobin A1c, examining feet annually, getting eyes dilated, checking blood pressure, and visiting the doctor annually. Sample demographics by sex were assessed. Unadjusted analyses were computed for descriptive statistics by sex and proportions of QOC indicators over time. Logistic regression evaluated associations between QOC indicators and sex, while controlling for sociodemographic characteristics, time, and comorbid conditions.

**Results:**

Approximately 44% and 56% of the sample was comprised of men and women, respectively. Unadjusted analyses showed significant differences in A1c testing (*p* < 0.001) and foot examinations (*p* = 0.002) for the entire sample, and significant differences in A1c testing (*p* = 0.027), foot examinations (*p* = 0.01), and dilated eye exams (*p* = 0.026) among men and A1c testing (*p* < 0.001) among women overtime. Adjusted analyses found women to be significantly more likely to have dilated eye examinations during a given year (OR = 1.14; 95% CI 1.04, 1.24), to get their blood pressure checked by a doctor in a given year (OR = 1.44; 95% CI 1.13, 1.84), and to visit a doctor annually (OR = 1.39; 95% CI 1.22, 1.58) compared to men.

**Conclusions:**

In this sample of adults with diabetes, women had significantly higher odds of receiving quality of care compared to men. These findings suggest the importance of educating patients about appropriate metrics of diabetes management, especially men, and the need for continuous empowerment of women to receive proper and optimal care. Additional research is needed to identify causes and reduce sex and gender disparities associated with diabetes quality of care.

## Background

More than 29 million people in the United States are living with diabetes; of which, one in four are unaware they have diabetes [1]. Diabetes results in significant morbidity and mortality associated with complications such as adult-onset blindness, kidney failure, heart disease, stroke, and loss of limbs [1]. In 2010, 60% of non-traumatic lower-limb amputations occurred in patients with diabetes, and in 2011, diabetes was the number one cause of kidney failure [1]. In addition to its association with serious and adverse complications, diabetes also accounts for a tremendous financial burden to diagnosed patients and an economic burden to society. Evidence suggests women typically have higher healthcare costs for diabetes care compared to men [2], spending approximately $8,331 annually compared to $7,458 for men [2, 3]. The average cost of diabetes care for the U.S. is $245 billion, with 43% of that cost going towards hospital inpatient care [3]. Since 2007, the cost of diabetes care has risen 41%, yet the disease remains the seventh leading cause of death in the U.S. [1, 2, 4].

Over 15 million men and 13 million women have diabetes [1]. Sex and gender are important characteristics to consider in diabetes care as predisposition and exposure to multiple genetic, biological, cultural, environmental, and psychosocial risk factors, among others, contribute to differences in outcomes between men and women [5, 6]. For example, in women with diabetes, evidence suggests an increased risk for cardiovascular disease and mortality compared to women without diabetes and men, in part due to alterations in and lower levels of high-density lipoprotein cholesterol (HDL-C) and its associated lipid transfer proteins and enzymes, all of which protect against the development of atherosclerosis [5]. In addition, endocrine imbalances and psychosocial stress are associated with poorer outcomes in women with diabetes compared to men with diabetes [6]. Finally, lifestyle and behavioral factors contribute considerably to sex and gender differences in diabetes outcomes [6]. Recognizing, understanding, and being aware of differences between men and women with diabetes argues the relevance of considering sex and gender when managing diabetes and providing clinical care.

It is well known that quality care and optimal management in diabetes require a multifaceted and comprehensive approach. Quality of care (QoC) in patients with diabetes is of paramount importance and can be assessed using multiple performance indicators and process and quality measures such as testing the glycosylated hemoglobin A1c (HbA1c), checking the blood pressure (BP), examining the feet, receiving dilated eye examinations, and visiting the doctor. According to the American Diabetes Association (ADA), HbA1c testing should be performed at least 2-4 times a year depending on whether specific treatment goals are being met [4]. Additionally, to manage cardiovascular risk factors, the ADA recommends blood pressure be measured at each routine visit [4]. Similarly, a comprehensive foot evaluation and eye examinations are recommended to identify risk factors for ulcers and amputations and retinopathy, respectively [4]. These processes of care, along with others such as periodic testing of lipids and albumin, aspirin use, and smoking cessation, are needed to favorably affect health outcomes by achieving glycemic control, preventing the development of long-term complications, and reducing the financial burden associated with diabetes management [4, 7–10].

Compared with studies that assess differences in cardiovascular risk factors among adults with diabetes, less attention has been given to examining differences in receipt of QoC indicators within the same population [11–17]. Given observed differences between men and women with diabetes in risk factor control for cardiovascular disease, it is important to understand potential differences in how processes of care are received to understand the current climate of practice and establish new premises for research, policies, and clinical care. Therefore, in this study, we examined sex differences in receiving QoC indicators among a nationally representative sample of adults with diabetes. Based on evidence from the current literature, we hypothesized that women with diabetes would receive more QoC indicators compared to men with diabetes.

## Methods

### Data source and study population

The Medical Expenditure Panel Survey Household Component (MEPS-HC) data from 2002–2011 were used to examine the association between QoC and sex among adults with diabetes (aged ≥18 years). In this retrospective study, we identified 17,702 (weighted sample of 17,857,174) adults with self-reported diabetes from MEPS-HC. MEPS is a survey of a nationally representative U.S. civilian, non-institutionalized population and is administered by the Agency for Healthcare Research and Quality [18–20]. The AHRQ validates MEPS as a self-reported instrument by administering many quality assurance procedures like validation of an interviewer’s work and also comparing MEPS numbers with other data source numbers like the Census Bureau and National Health Interview Survey (NHIS). MEPS obtains information on participants’ quality of care, as well as information on medical spending, demographics, and socioeconomics [20]. The MEPS sample is drawn from reporting units in the previous year’s NHIS, a nationally representative sample with oversampling for non-Hispanic Blacks and Hispanics of the U.S. civilian, non-institutionalized population [18–20].

To ensure sufficient sample size and robust estimation for our analyses, we pooled 10 years of MEPS data. Because they have a common variance structure, we can ensure compatibility and comparatively of our variables within the complex sample design. Our study accounts for the sampling weights, clustering, and stratification design to estimate the nationally representative study for the U.S. population [20].

### Measures

#### Variables of interest

All measures are based on previously validated questionnaires that are publicly available on the MEPS website [18–20]. The dependent variables in this study were five binary indicators to measure the QoC for patients with diabetes, which reported receipt of the following during the past year: 1) having at least two HbA1c tests, 2) having at least one foot examination, 3) having a dilated eye examination, 4) having blood pressure checked by a doctor, and 5) having at least one visit to the doctor’s office for care. The primary independent variable was sex, which was dichotomized as female versus male.

### Controlled covariates

All controlled covariates used for analysis were based on self-report. Binary indicators of comorbidities were based on a positive response to a question, “Have you ever been diagnosed with…?” Cardiovascular disease (CVD) indicates a positive response to coronary heart disease, angina, myocardial infarction, or other heart diseases. Race/Ethnic groups were categorized into: Non-Hispanic White (NHW), Non-Hispanic Black (NHB), Hispanic, or others. Education was categorized into: less than high school (≤grade 11), high school (grade 12), and college or more (grade ≥13). Marital status was categorized into: married, non-married, and never married. Age was categorized into: 18–44, 45–64, and ≥65 years. Metropolitan Statistical Area (MSA) was coded by MSA status (MSA = 1, Non-MSA = 0). Census region was categorized into: Northeast, Midwest, South, and West. Health insurance was categorized into: private, public only, and uninsured throughout the year. The income level was defined as a percentage of the poverty level and grouped into four categories: poor (<125%), low income (125% to less than 200%), middle income (200% to less than 400%), and high income (≥400%). Calendar year was grouped into three consecutive years of 2002/05, 2006/09, 2010/11 for the pooled data.

### Analysis

The baseline characteristics of adults with diabetes are presented by sex, with percentage differences for categorical variables tested using *χ* ^2^ tests. Bivariate analyses were used to compare the trends of receiving QoC and then trends by sex. We also used multiple logistic regression to examine factors associated with sex and receiving QoC variables. Models controlled for age, race/ethnicity, marital status, education, insurance status, MSA, census region, household income, comorbidities, and time trend. For interpretation, we use the odds ratio coefficient of the logistic regressions.

F-adjusted mean residual goodness-of-fit was applied to test the adequacy of the models. After fitting the logistic regression models, taking the survey design into account, the F-adjusted mean residual goodness-of-fit suggested no evidence of lack of fit [21]. We used the link test that considers complex survey design as a diagnostic test to examine the model specification error. We verified the evidence of proper specification of the model [22, 23]. Using the Variance Inflation Factor (VIF) test, it was determined that no multicollinearity problems existed between predictors of the model. We performed all analyses at the person-level using Stata 14 [24]. Estimates that are statistically significant at the *p* < 0.05 level are discussed in the paper.

## Results

### Characteristics of U.S. adults with diabetes population

Characteristics of adults with diabetes in this study are summarized in Table 1. The unweighted sample size was 17,702 for adults with diabetes (aged ≥18 years) and represented a weighted sample of 17,857,174 within the U.S. population. Of the unweighted sample, 56.4% were women and more likely to be in age group 18–44 years and elderly (≥65 years), be minority, single, have a lower education level, be publicly insured, have poor and low income, and have a comorbid condition of cardiovascular disease, asthma, joint pain and arthritis.

**Table 1:** Sample demographics by sex among adults with diabetes

	All (*n* = 17,702)	Men (*n* = 7715)	Women (*n* = 9987)	*P*-value
Age				<0.001***
18–44 years	13.2	12.2	14.1	
45–64 years	46.9	49.6	44.4	
65+ years	39.9	38.2	41.5	
Race/Ethnicity				<0.001***
Non-Hispanic White	64.6	68.4	61.0	
Non-Hispanic Black	15.3	12.3	18.0	
Hispanic	13.5	12.7	14.3	
Non-Hispanic Other	6.6	6.6	6.7	
Marital Status				<0.001***
Married	58.5	70.1	47.4	
Unmarried	32.5	21.7	42.8	
Never married	9.1	8.3	9.8	
Educational Level				<0.001***
<High School Graduate	26.9	24.1	28.2	
High School Graduate	34.5	32.1	36.9	
College Graduate	39.3	43.9	35.0	
Insurance Status				<0.001***
Private Insurance	61.0	66.2	55.97	
Public insurance	31.5	26.1	36.6	
No Insurance	7.6	7.7	7.4	
Annual Family Income Level				<0.001***
Poor/Negative/Near Poor Income	19.9	15.1	24.6	
Low Income	16.2	14.1	18.3	
Middle Income	30.8	31.2	30.3	
High Income	33.1	39.6	26.8	
Metropolitan Statistical Area				.101
Metropolitan Statistical Area	79.7	79.0	80.5	
Non-Metropolitan Statistical Area	20.3	21.0	19.6	
Region				0.556
Northeast	17.2	17.3	18.1	
Midwest	21.1	21.2	21.1	
South	40.3	40.1	40.5	
West	20.8	21.4	20.2	
Hypertension				0.126
No	26.8	27.7	26.0	
Yes	73.2	72.3	74.0	
Cardiovascular Disease				0.001**
No	68.1	66.3	69.8	
Yes	31.9	33.7	30.2	
Stroke				0.819
No	89.9	90.0	89.8	
Yes	10.1	10.0	10.2	
Emphysema				0.266
No	95.1	94.8	95.4	
Yes	4.9	5.2	4.7	
Asthma				<0.001***
No	86.3	90.6	82.2	
Yes	13.7	9.4	17.8	
Joint Pain				<0.001***
No	43.8	49.0	38.8	
Yes	56.2	51.0	61.2	
Arthritis				<0.001***
No	51.2	59.8	42.9	
Yes	48.8	40.2	57.1	
Year Categories				0.511
2002–2005	33.9	33.3	34.4	
2006–2009	42.6	42.8	42.4	
2010–2011	23.5	23.9	23.0	

*n* = unweighted sample size. All numbers represent percentages

Statistically significant at *p < 0.05, **p < 0.01, and ***p < 0.001

### Quality of care changes over time

Among adults with diabetes, receiving an HbA1c test at least twice during the year and receiving at least one foot examination during the year were significantly associated with time trend (Table 2). Receiving an HbA1c test at least twice during the year rose consistently from 78.1% in 2002/05 to 83.5% in 2010/11. Receiving at least one foot examination during the year decreased from 71.2% in 2002/05 to 67.2% in 2006/09 and then rose to 69.6% in 2010/11.

**Table 2:** Unadjusted proportions for quality of care indicators overtime among adults with diabetes

Quality of care indicators	2002–2005	2006–2009	2010–2011	*P*-value
HbA1c test 2+ times during year?	78.1	80.0	83.5	<0.001***
Feet checked during year?	71.2	67.2	69.6	0.002**
Dilated eye exam during year?	61.9	62.5	62.9	0.063
Blood pressure checked by doctor during year?	97.8	97.9	98.1	0.648
Visited doctor during last 12 months?	91.2	91.0	90.3	0.455

All numbers represent percentages. Statistically significant at *p < 0.05, **p < 0.01, and ***p < 0.001

Quality of care indicators by sex overtime among adults with diabetes is shown in Table 3. Receiving an HbA1c test at least twice during the year was significantly associated with time trend, and it consistently increased overtime for both men and women. Having a dilated eye exam and having at least one foot examination during the year were significantly associated with time trend for men only. Having at least one foot examination in men decreased from 72.3% in 2002/05 to 67.9% in 2006/09 and then increased to 71.3% in 2010/11. Having a dilated eye exam in men consistently increased from 61.0% in 2002/05 to 65.9% in 2010/11.

**Table 3:** Descriptive statistics for quality of care indicators by sex overtime among adults with diabetes

Quality of care indicators	Men				Women			
	2002–2005	2006–2009	2010–2011	*P*-value	2002–2005	2006–2009	2010–2011	*P*-value
HbA1c test 2+ times during year?	77.9	80.4	82.6	0.027*	78.3	79.6	84.3	<0.001***
Feet checked during year?	72.3	67.9	71.3	0.010*	70.1	66.8	68.0	0.089
Dilated eye exam during year?	61.0	61.5	65.9	0.026*	62.6	63.5	64.3	0.631
Blood pressure checked by doctor during year?	97.2	97.9	97.7	0.323	98.3	97.9	98.5	0.177
Visited doctor during last 12 months?	90.1	90.2	88.7	0.326	92.1	91.8	91.9	0.904

All numbers represent percentages. Statistically significant at *p < 0.05, **p < 0.01, and ***p < 0.001

### Receipt of quality of care

In the fully adjusted multiple logistic regression model (Table 4), women had statistically significant higher odds of receiving a dilated eye exam compared to men (OR = 1.14; 95% CI 1.04–1.24). Women had statistically significant higher odds of getting their blood pressure checked by a doctor during the year (OR = 1.44; 95% CI 1.13–1.84) in comparison to men. Compared to men, women had significantly higher odds of making at least one visit to the doctor for care (OR = 1.39; 95% CI 1.22–1.58). Sex was not significantly associated with higher or lower odds of having HbA1c tested at least twice or having at least one foot examination during the year.

**Table 4:** Adjusted logistic regression for quality of care indicators among adults with diabetes

Quality of care indicators	Odds ratio	95% Confidence interval	*P*-value
HbA1c test 2+ times during year?	1.01	(0.89, 1.14)	0.848
Feet checked during year?	0.91	(0.83, 1.00)	0.066
Dilated eye exam during year?	1.14	(1.04, 1.24)	0.003**
Blood pressure checked by doctor during year?	1.44	(1.13, 1.84)	0.004**
Visited doctor during last 12 months?	1.39	(1.22, 1.58)	<0.001***

Statistically significant at *p < 0.05, **p < 0.01, and ***p < 0.001. Reference Group: men

Adjusted for: age, race/ethnicity, sex, marital status, education, insurance, income, metropolitan statistical area, region, income, comorbid conditions (hypertension, cardiovascular disease, stroke, emphysema, asthma, joint pain, and arthritis) and time

## Discussion

In this sample of adults with diabetes, women had significantly higher odds of receiving certain QoC indicators compared to men after adjusting for confounding factors. Specifically, women were significantly more likely to visit a doctor, and receive blood pressure checks and eye examinations in comparison to men with diabetes. These findings suggest that, compared to men with diabetes in this national sample, women were more likely to receive QoC processes of care. Given the cross-sectional design of this study, definitive causes for this difference between men and women cannot be identified; however, it is possible that women had higher odds of receiving QoC because of their increased rate of utilization, desire for preventive healthcare services, higher prevalence of comorbid conditions, and longer life expectancies [16, 25–28].

Our findings are supported by evidence from previous studies evaluating differences in QoC indicators between men and women with diabetes. In this sample of adults, we found women to have higher odds of visiting a doctor annually and receiving QoC indicators such as blood pressure measurements and dilated eye examinations. This is similar to the findings of Chou et al and Tseng et al, who also found women more likely to receive certain performance indicators such as eye examinations [12, 15]. Similarly, among adults enrolled in managed care plans, a sex disparity was observed where women with diabetes, again, had a higher relative risk for receiving eye examinations compared to men [17]. Additionally, in our study, we did not find sex differences in HbA1c testing for QoC indicators. This is similar to the findings of Yu et al [14] and Tseng et al [15], who found no sex differences in HbA1c testing among adults with diabetes. In contrast to our findings, there is evidence to support poorer QoC in women with diabetes compared to men, despite QoC being a priority for most women [11, 13, 14, 17, 29]. For example, in a cross-sectional study to assess gender differences across racial and ethnic groups in QoC for diabetes, Correa-de-Araujo and colleagues found NHW women less likely to receive retinal eye and foot examinations than NHW men [11]. Evidence suggests several reasons women possibly have higher risks of not reaching targeted recommendations compared to men; these include 1) lower income and education in women, 2) opposing lifestyles, attitudes, and beliefs, 3) missed clinical goals, particularly for BP, and low-density lipoprotein-cholesterol (LDL-C) and even for composite control of BP, HDL-C, LDL-C, and HbA1c, 4) differences in rates of modifiable risk factors, and 5) lower screening rates for women compared to men [11, 13, 14, 17, 29, 30]. Lastly, some studies have found no differences in QoC indicators between men and women; however, recommendations were noted that improvements in QoC are needed overall for all adults [11, 12, 14, 29, 31].

The findings of our study are important because they reemphasize the importance of improved QoC for both men and women with diabetes. In the nation’s health agenda to reduce disparities and improve the health of all Americans, *Healthy People 2020* shared recommendations for certain QoC that must be received by patients with diabetes [32]. Despite prior evidence from the remote and recent past showing progress in QoC trends for patients with diabetes [32–34], many adults are still not reaching recommended goals for diabetes QoC. For example, except for HbA1c testing at least twice a year and annual dilated eye examinations, adults with diabetes (≥18 years of age) did not meet other *Healthy People 2020* recommendations for preventive care practices [32]. This trend has also been acknowledged in the literature [35-36]. A study conducted in patients with diabetes found that only 14% of physicians document proper foot examinations on their patients [35]. To complicate this issue, it is often reported that primary care physicians do not have the time or training to properly educate patients with diabetes on appropriate self-care of their feet [36]. In addition to difficulties meeting preventative care goals, it has been reported that, between 2009 and 2012, 71% of adult patients with diabetes did not meet recommended goals for blood pressure [4].

While this study adds to the current literature assessing disparities in QoC between men and women with diabetes, there are limitations that must be reported. First, cross-sectional studies are limited in being able to draw causal associations. Second, consistent quality indicators and approaches for measuring QoC in diabetes are lacking in the current literature, where the focus was often on intermediate outcomes such as goal attainment (i.e., HbA1c < 7%, systolic and diastolic BP < 140/90 mmHg, LDL-C < 100 mg/dL, urine microalbumin, etc.) in different combinations, and not solely on receipt of services. Because of these varied approaches, it was difficult to obtain a comprehensive assessment of QoC for adults with diabetes. In addition, performance on other quality, process, and intermediate indicators such as LDL-C, smoking, obesity, albumin-creatinine ratio, and estimated glomerular filtration rate (eGRF) were not included in these analyses. Third, when adjusting for sociodemographic characteristics, comorbid conditions, and time trend in the analyses, adjustments were not made for potential confounding variables such as racism and perceived discrimination, patient-provider communication and attitudes, and dissatisfaction with care, all of which may have influenced patient utilization and patient-provider decision-making. Fourth, given the design of MEPS data, it is not possible to distinguish between type 1 and type 2 diabetes, limiting analyses on sex-related differences in the QOC indicators by type of diabetes diagnosis. Therefore, the findings reported herein indicate differences by sex for all individuals with diabetes, regardless of type.

## Conclusions

The results of our study are important and provide additional information about sex differences in specific QoC indicators among adults with diabetes. In this nationally representative sample of men and women with diabetes, women had higher odds of visiting a doctor, and receiving BP checks and eye examinations compared to men after adjusting for relevant confounding factors. Despite women performing better for specific QoC indicators, more progress is needed for both men and women to meet recommended targets.

Overall, these results indicate a need for improved QoC in adults with diabetes, as well as a need for uniformity in constructs and quality indicators and assessments to measure QoC in diabetes care. Additional research is needed to assess differences in QoC and to identify facilitators and barriers to appropriate QoC between men and women with diabetes. Policies are needed that propose strategies for addressing disparate care between men and women with diabetes. Suitable sex-specific guidelines are warranted in clinical practice for minimizing suboptimal QoC in adults with diabetes and increasing the number of adults who meet QoC targets and recommendations. Finally, it is important that providers discuss QoC processes with their patients to educate and inform them about appropriate diabetes management, empowering them to participate in the decision-making processes to ensure QoC indicators are received and targeted goals met.

## Abbreviations

ADA: American diabetes association
AHRQ: Agency for healthcare research and quality
BP: Blood pressure
CVD: Cardiovascular disease
HbA1c: glycosylated hemoglobin A1c
HDL-C: High-density lipoprotein-cholesterol
LDL-C: Low-density lipoprotein-cholesterol
MEPS: Medical expenditure panel survey
MEPS-HC: Medical expenditure panel survey household component
mg/dL: milligrams per deciliter
mmHg: millimeters of mercury
MSA: Metropolitan statistical area
NHB: Non-hispanic black
NHIS: National health interview survey
NHW: Non-hispanic white
QoC: Quality of care
U.S.: United States
